# Experimental Study on Properties of Modified City Wall Soil

**DOI:** 10.3390/ma15238615

**Published:** 2022-12-02

**Authors:** Wei Pan, Jianwei Yue, Xue Yang, Zifa Wang

**Affiliations:** 1 Henan Communication Vocational Technology College, Zhengzhou 450000, China; 2 School of Civil Engineering and Architecture, Henan University, Kaifeng 475004, China

**Keywords:** disintegration test, shear strength, specific absorption of quality, micro analysis

## Abstract

Due to the special geographical location, climatic characteristics, special soil properties and the flood zone of the Yellow River in Kaifeng, the groundwater level in the lower reaches of the river basin is high and contains much salt. The matrix suction and surface free energy of the Kaifeng city wall earthen site changed under capillary action, resulting in cracking, peeling and efflorescence to varying degrees. In order to reduce the deterioration of the Kaifeng city wall caused by environmental erosion, a select lime with excellent mechanical properties and waterproof methanesiliconic acid sodium salt with excellent water resistance were chosen to reinforce the earthen sites. In this paper, 0%, 3%, 5% and 7% lime, and 0%, 1%, 2% and 3% waterproof materials were selected to determine four types of imitation site soil with 16 different mix proportion samples. Further, samples with different mixing ratios were subjected to direct shear, a disintegration test, and a microscopic scanning electron microscope test. The results show that under different normal stresses, with the increase in waterproof material content, the growth rate of shear strength of imitation site soil ranges from 1.82% to 10.81%. With the increase in lime content, the shear strength of imitation site soil increases rapidly, up to 38.16%. Both materials can improve the shear strength of the soil site. Under reinforcement with the two materials, the cohesive force of the imitation site soil can be improved at a maximum rate of 59.23%, and the internal friction angle changes in the range of 36.72°–41.61°. Compared with the sample without waterproof material, the mass water absorption rate of the sample with waterproof material decreases in the range of 2.76–27.77, and with the increase in waterproof material, the mass water absorption rate of the sample gradually decreases. The chemical reaction products of the waterproof materials and lime can play a filling role in silty clay; filling micro-pores and micro-cracks between soil particles.

## 1. Introduction

**Figure 1:**
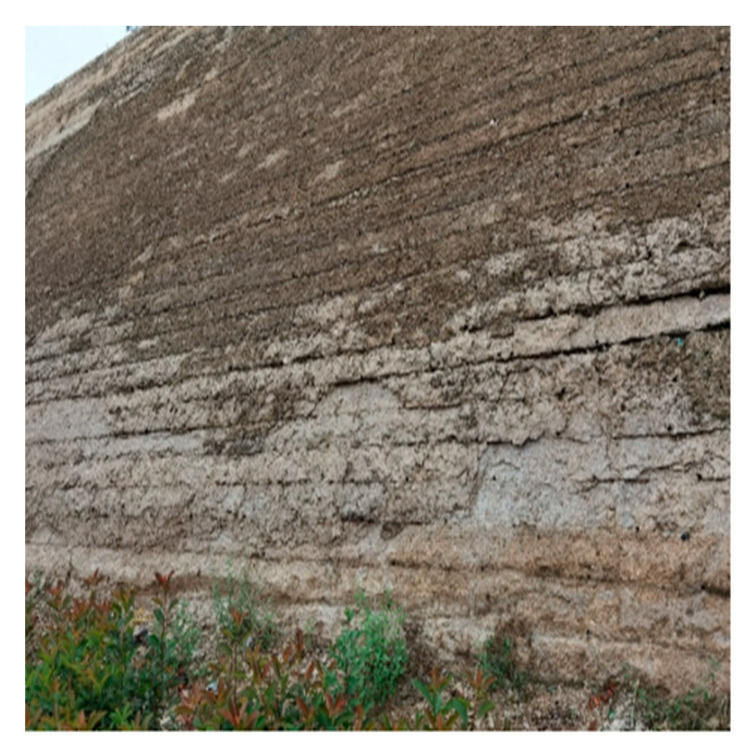
Deterioration of Kaifeng city wall.

Most of the earthen sites in Henan Province are open-air, above-ground and alternating wet and dry nature sites; they are precious immovable cultural relics with high historical value, such as the Kaifeng city wall earthen sites (Figure 1), city stacking earthen sites, Zhengzhou Mall sites, Qingliangsi Ruguan kiln sites, etc. Due to the complex impact of the construction process and the environment in which they have been located for a long time, these earthen sites experience alkali, flaking, cracks and even collapse phenomenon [1,2,3]. Taking the Kaifeng city wall earthen site as an example, most of the existing walls were built during the Qing Dynasty, and the environment in which they are located has relatively abundant surface water resources and have suffered from floods many times in history [4]. Rainfall can lead to the deterioration of soil sites [5,6], and Kaifeng has more concentrated rainfall in summer; further, the body of these soil sites are mostly powdered clay, which is prone to water absorption and weathering, vigorous capillary action, and rapid salt transport. Soluble salts accompanied by capillary used in the walled soil sites, constantly migrate and crystallize, resulting in changes in the internal structure of the Kaifeng walled soil sites under capillary action, and showing different degrees of cracking, spalling and alkali flooding. Thus, it is particularly important to select reinforcement materials that improve the mechanical properties of the soil site while effectively addressing the deterioration of the soil site by water.

In recent years, domestic and foreign scholars have conducted extensive research on materials that can be used to reinforce earthen sites, including lime, glutinous rice slurry, and microbial mineralization, which are effective in resisting weathering and improving mechanical properties [7,8,9,10]. In early human construction activities, lime was one of the first inorganic cementitious materials used; it had good permeability and air permeability and was compatible with ancient building matrix materials [11,12]. In order to achieve better results when repairing the soil of the site, lime was also used to reinforce the earthen site to give it better strength and stability [1,13,14,15]. The research results of later scholars have contributed to the improvement of soil sites, including the improvement of their mechanical properties; however, there are few studies that have examined how both the mechanical properties and the hydraulic properties of soil sites can be improved. In this paper, we use methosilicate waterproofing materials and lime as reinforcement materials. Methosilicate waterproofing materials are widely used in the fields of waterproofing, hydrology, and material protection [16,17,18], as they have strong waterproofing, weathering resistance, and mechanical properties; however, there are few studies in the field of site soil protection. When lime is used as a repair material, it can react with water and CO_2_ to generate Ca(OH)_2_ and CaCO_3_; thus, enhance the cohesion between soil particles.

In this paper, we used a combination of organic material lime and a methyl silicic acid type waterproof agent to restore the site of the Henan section of the Yellow River Basin, and investigated the mechanism by which improvement of the mechanical and hydraulic properties of the Kaifeng city wall was achieved using indoor direct shear tests, a disintegration test, and microscopic analysis. The research methods and results will provide new ideas for moisture proofing and waterproofing of earthen sites and ancient city wall foundations in the Henan section of the Yellow River Basin.

## 2. Test Materials and Test Protocol

### 2.1. XRF Analysis of Soil Samples

**Figure 2:**
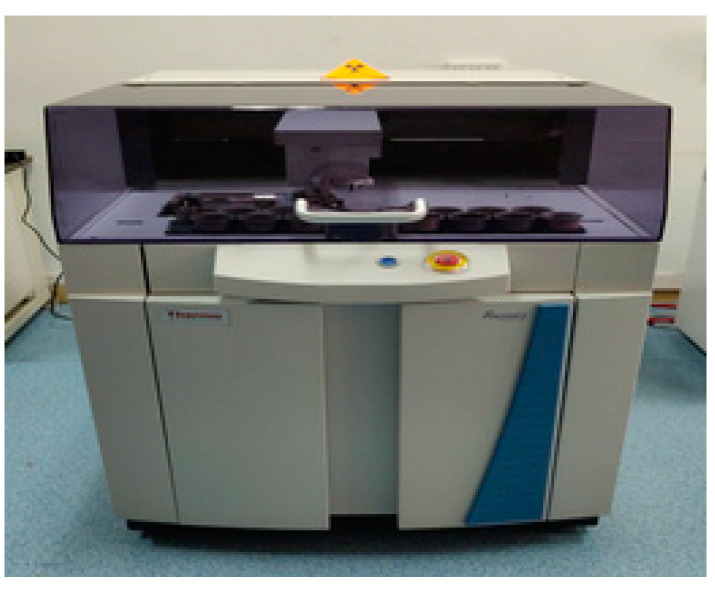
X-ray fluorescence spectrometer.

**Figure 3:**
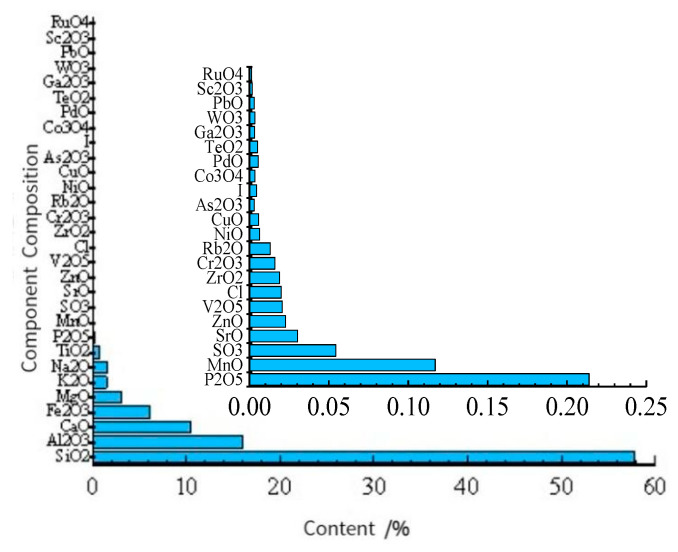
Element content analysis results of silty clay by X-ray fluorescence spectrometry.

The working principle of the X-ray fluorescence analysis method is that the incident X-ray interacts with the substance to produce the characteristic X-ray fluorescence intensity. According to the relationship between the fluorescence intensity and the content of the elements to be analyzed, the content of each element to be measured can be obtained by using the corresponding working curve. X-ray fluorescence sample processing does not require wet digestion. The spectral lines are very simple. More than 90 kinds of elements can be analyzed, and no pollution will occur after the sample measurement. Many elements can be determined. It has many advantages, such as rapid analysis, no damage to the sample attributes, a wide analysis range, stable and reliable results, rapid simultaneous analysis of multiple elements, simple operation, etc. The equipment used in this section is the X-ray fluorescence spectrum analyzer (ARLAdvantX Intellipower TM3600) produced by the Thermo Flying Company, as shown in Figure 2. The analysis results are shown in Table 1 and Figure 3.

It can be seen from Table 1 and Figure 3 that the main components of the selected silty clay are SiO_2_, Al_2_O_3_, CaO, Fe_2_O_3_ and MgO, and their mass proportions are 57.79%, 16.05%, 10.52%, 6.16% and 3.11%, respectively. The main elements are Si, Al, Ca, Fe and Mg, and their mass proportions are 27.02%, 8.49%, 7.52%, 4.31% and 1.89%, respectively. As other substances and elements only account for a small proportion, they are not included in the calculation and research.

### 2.2. Test Material

**Figure 4:**
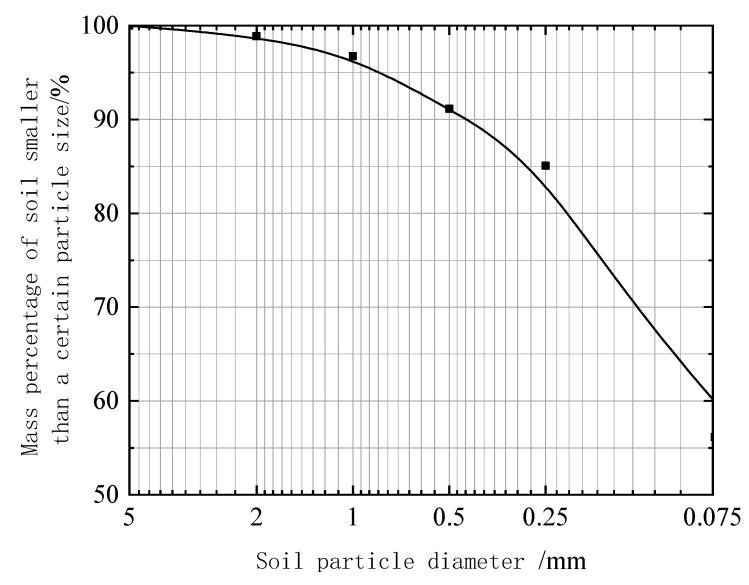
Sieving curve of powdered clay particles.

A powder clay with similar basic physical properties to those of the Kaifeng city wall was selected for the Limit water content test to obtain its basic physical properties. The plastic limit was 21.23%, the liquid limit was 37.33%, the plasticity index was 16.1, the maximum dry density was 1.68 g/cm^3^, and the optimum moisture content was 14.32%. The particle sieving test was carried out by using an LP-100D digital display soil liquid plastic limit tester, and the gradation of the four parallel groups of specimens was calculated to be basically the same; the test results are shown in Figure 4.

Lime was purchased from Tianjin Comio Chemical Reagent Co., Ltd. and the main physicochemical indexes are as follows. The relative molecular mass is 56.08, CaO content is greater than or equal to 98%, cauterization loss is 2%, and the state is powder.

The waterproof material selected was a methyl silicic acid type waterproof agent; a colorless transparent liquid, soluble in water, having good waterproof and weathering resistance itself, and widely used in waterproofing, moisture and material protection, etc.; the pH value was between 12–14.

### 2.3. Specimen Preparation

**Figure 5:**
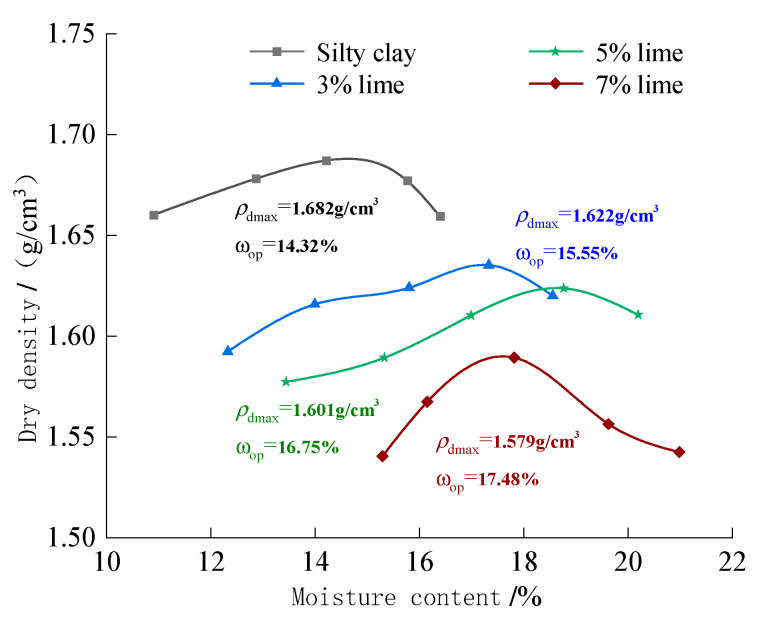
Dry density and water content curve.

Lime can chemically react with H_2_O and CO_2_, and consume a part of pure water, which has a great influence on the water content. Therefore, compaction tests were conducted on soil materials mixed with 0%, 3%, 5% and 7% lime, and three parallel specimens were set for each mixture. The results of the compaction test are shown in Figure 5, and the test results are in accordance with the general rule that there is a peak in the compaction curve, and the greater the lime content, the smaller the dry density [19], and the greater the corresponding optimum moisture content [20]. The test results show that the compaction test results are related to the amount of lime mixture.

The retrieved powdered clay was dried, crushed, sieved to remove impurities and prepared for use. After the pre-test trial, we selected 0%, 3%, 5% and 7% of lime, and 0%, 1%, 2% and 3% of waterproofing materials, to determine the four types of sixteen different ratios of specimens of imitation site soil; the specific formula is shown in Table 2. Using the optimum moisture content and maximum dry density obtained from the compaction test, the pulverized clay, lime, and waterproofing materials were mixed with pure water in proportion, mixed well, and sealed and left to stand for 24 h. The specimens for the direct shear test (61.8 mm × 20 mm) and disintegration test (39.1 mm × 80 mm) were prepared by using a ring knife and triple flap membrane, and three parallel specimens were set up for each test scheme. The specimen preparations were placed in a constant temperature and humidity chamber for 28 days (temperature 20 °C, 90% relative humidity), so that the lime, waterproof materials and powdered clay fully reacted.

### 2.4. Test Method

#### 2.4.1. Direct Shear Test

For the direct shear test, an automatic straight shear instrument produced by Beijing Huakan Technology Co. was used in accordance with The Standard for Geotechnical Test Methods [21]. We set the shear rate to 0.8 mm/min, and applied vertical pressures of 50 kPa, 100 kPa, 150 kPa and 200 kPa to perform the direct shear test, and determined the shear strength, cohesion and internal friction angle of specimens with different ratios.

#### 2.4.2. Disintegration Test

**Figure 6:**
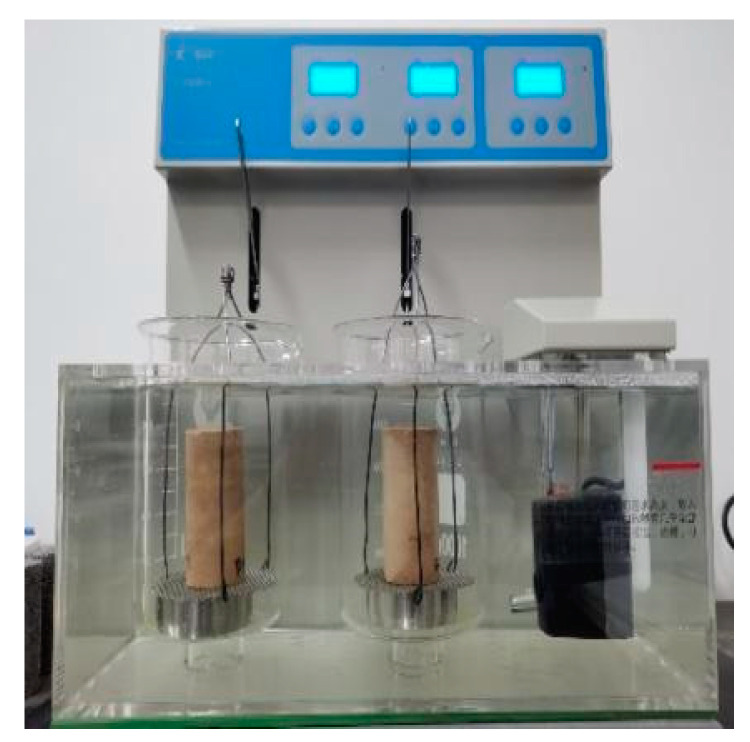
Double cup disintegrator.

The disintegration test complied with The Standard for Geotechnical Test Methods (GB/T50123-2019) [22] by using a double cup disintegrator. The test process can be filmed in real time and the mass of the specimen can be weighed. The specific test apparatus is shown in Figure 6. The outer layer of the apparatus is made of Plexiglas and the inner layer consists of a beaker, wire mesh (10 cm in diameter), ring knife (6.18 cm in diameter) and steel wire. The water temperature can be controlled by injecting water between the outer layer and the inner layer. The soil sample of the improved site is placed on the hanging net and then immediately put into the inner beaker, and the camera recording device is turned on to record the wetting and disintegration of the specimen at each stage in real time; the wetting and disintegration of the specimen is recorded within 24 h.

#### 2.4.3. Microscopic Scanning Electron Microscopy Testing

This test was observed via scanning electron microscope test equipment used for the FEI Quanta 250 environmental scanning electron microscope, before filming the sample after maintenance sampling. We roughly cut out of the sample a side length of 5 mm cubic pieces, which were placed at a constant temperature of 105 °C and oven baked for 8 h until thoroughly dried. Then, conductive adhesive was used to paste the specimen on the copper table, before plating with conductive film, and applying a gold spraying treatment. 

## 3. Test Results and Analysis

### 3.1. Direct Shear Test Results

**Figure 7:**
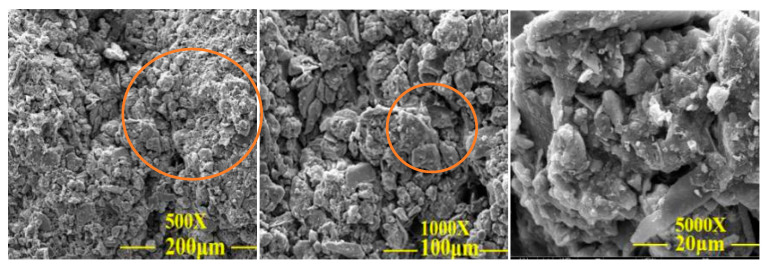
SEM of the plain soil group.

The shear strength of each ratio specimen under different normal stresses is shown in Figure 7. It can be seen from the figure that the shear strengths of the specimens with different ratio combinations under normal stresses of 50 kPa, 100 kPa, 150 kPa and 200 kPa increased to different degrees compared with A00. Lime, sodium methylsilicate and reaction products fill the space between soil particles, which improves the integrity of soil skeleton, compactly fills the soil particles, significantly improves the particle size distribution, has good cohesion between particles, and reduces the porosity of soil samples. The 50 kPa stress is in the range of 10.53–75%, 100 kPa stress in the range of 4.17–46.53%, 150 kPa stress in the range of 6.71–39.02%, and 200 kPa stress in the range of 10.29–56%. The overall increases in shear strength under different normal stress ratio specimens ranged from 4.17–75%. Different combinations of lime and waterproofing materials formed different mixes, resulting in a discrete shear strength improvement range. The preliminary lime and waterproofing materials needed to improve the effectiveness of the site soil shear strength is more obvious. When the lime content is 0%, the unconfined compressive strength of Kaifeng city wall restoration soil gradually increases with the increase in sodium methylsilicate content. When the sodium methylsilicate content is 1%, 2%, and 3%, the unconfined compressive strength is 11.69%, 17.61%, and 24.57% higher than A00, respectively. It can be seen that the unconfined compressive strength of Kaifeng city wall restoration soil can be improved by adding sodium methylsilicate alone. When the lime content is 3%, 5% and 7%, the unconfined compressive strength of Kaifeng city wall restoration soil is effectively improved to varying degrees. At the same lime content, the unconfined compressive strength of Kaifeng city wall restoration soil first increases and then decreases with the increase in sodium methylsilicate content. When the sodium methylsilicate content exceeds 2%, the unconfined compressive strength of Kaifeng city wall restoration soil decreases. The reason is that both lime and sodium methylsilicate need to react with carbon dioxide in the air. When the concentration of carbon dioxide in the curing conditions is insufficient, the reaction between lime and sodium methylsilicate will be uneven, which affects the improvement in the mechanical properties of Kaifeng city wall repair soil. This is because the Ca (OH)_2_ crystals and CaCO_3_ precipitates generated by the chemical reaction of lime can fill the pores between soil particles, reduce the porosity between soil particles, and increase the compactness of the Kaifeng city wall repair soil. Sodium methylsilicate can fully react with water and CO_2_ to form enough polysiloxane films.

When the amount of lime is constant, the shear strength of the imitation site soil gradually increases with the increase in the amount of waterproofing material; the growth rate is in the range of 1.82–10.81% and it can be judged, again, that the waterproofing material is effective in improving the shear strength of the imitation site soil. The waterproofing material used has excellent mechanical properties and can improve the overall stability of the soil sample, which is consistent with the results obtained by relevant researchers [23]; when the amount of waterproofing material is constant, with the increase in the amount of lime, the shear strength of the imitation site soil increases significantly, with a maximum growth rate of 38.16%. Thus, it can be shown that both lime and waterproofing materials are beneficial for the improvement of the shear strength of the soil site, and the lime effect is greater than that of the waterproof material.

This is because the Ca(OH)_2_ crystal and CaCO_3_ precipitation generated by the chemical reaction of lime in the sample can greatly improve the shear strength, cohesion and internal friction angle of the sample.

**Figure 8:**
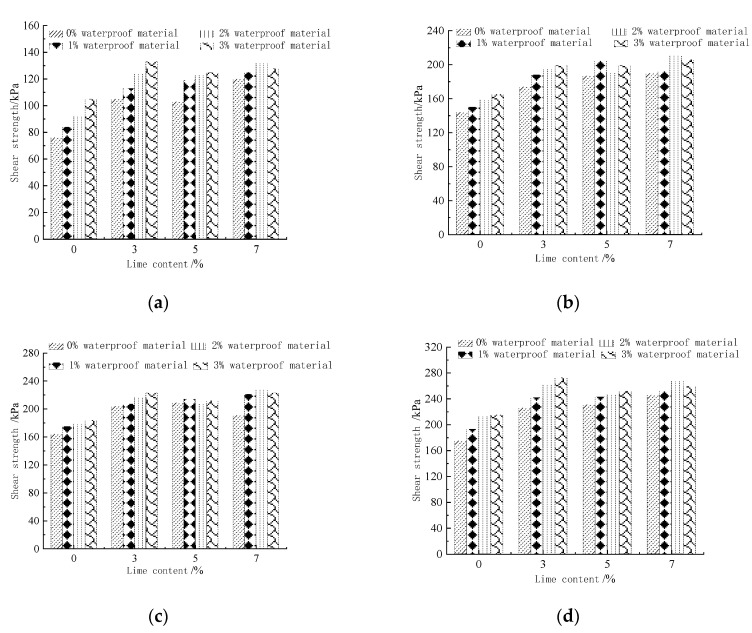
Shear strength of specimens of each ratio under different normal stresses. (**a**) Shear strength of different ratios at 50 kPa. (**b**) Shear strength of different ratios at 100 kPa. (**c**) Shear strength of different ratios at 150 kPa. (**d**) Shear strength of different ratios at 200 kPa.

Figure 8 shows the relationship curve between the shear strength index of the imitation site soil and the amount of reinforcement materials. It can be seen that, with the increase in lime and waterproofing material, the overall trend of the cohesion and internal friction angle of the imitation site soil increased. In addition, when the amount of lime is 3%, 5% and 7%, the cohesion and internal friction angle of the imitation site soil has a greater increase compared with the plain soil. When the amount of waterproofing material is 2%, and lime is 7%, the rate of increase in the cohesion of the imitation site soil reached the maximum rate of 59.23%. When lime and waterproofing material improves the imitation site soil at the same time, the internal friction angle of the imitation site soil is in the range of 36.72°–41.61°; the magnitude of change is small. However, with the increase in the amount of waterproofing material, the growth rate shows a trend of rapid increase followed by a slow decline. The reason is because both lime and waterproofing materials need to react with water and carbon dioxide in the air; when there is insufficient carbon dioxide in the maintenance environment, this leads to an incomplete chemical reaction between the lime and waterproofing material, affecting the improvement of the shear strength index of the imitation site soil. It can be seen that both the waterproofing material and lime have the ability to improve the shear strength index of the imitation site soil; however, the ability of the waterproofing material to improve the mechanical properties of the imitation site soil is less effective than lime.

### 3.2. Disintegration Test Results

Disintegration test process: The specimen is completely placed in the hydrostatic water for wet disintegration, and the rate of disintegration of the specimen in the hydrostatic water is measured; the greater the rate of disintegration, the weaker the water corrosion resistance of the specimen’s dispersion performance—a specimen impacted by water erosion and latent corrosion is more serious [23,24].

**Figure 9:**
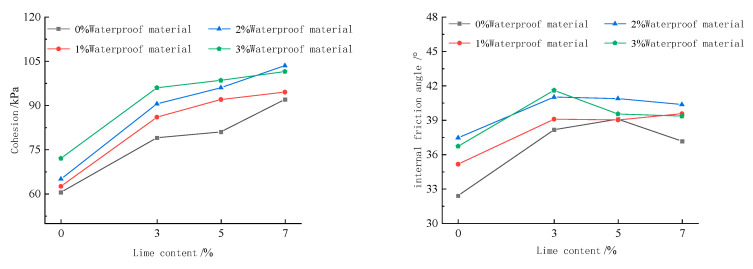
Relationship curve between the shear strength index of imitation site soil and the amount of reinforcing material admixture.

Figure 9 shows the appearance changes of each combination of the disintegration test after 24 h. A00 is the plain soil group, which disintegrated within a short time in the static water and, finally, became a pile of loose soil, with poor water stability and poor disintegration resistance; the disintegration rate could not be calculated by weighing its mass. Other ratio specimens experienced no disintegration phenomenon; the water stability and disintegration resistance was good, and during the disintegration test process, the slight water absorption caused by the quality of the specimen was not reduced but increased. The A01, A02, A03 specimens were only mixed with the waterproof material; the surfaces were smooth and complete, no disintegration phenomena occurred, and the water absorption phenomena were also not obvious, indicating that using waterproof materials to improve imitation site soil disintegration resistance and water stability is more effective. Waterproofing materials can be chemically reacted to form a hydrophobic polysiloxane film on the surface of the specimen and between the internal soil particles; water molecules cannot enter the internal pores of the specimen to resist capillary water on the specimen, improving the water stability of the imitation site soil. The A30, A50, A70 specimens were only mixed with lime; there were tiny bubbles in the disintegration test process, indicating that the specimens had a small amount of water absorption phenomena, but the overall disintegration of the specimens did not occur, and the disintegration resistance was good, and, thus, the disintegration resistance of A00 was improved. The Ca(OH)_2_ crystals and CaCO_3_ precipitation generated by the chemical reaction of lime can fill the pores between soil particles, reduce the porosity between soil particles, and increase the compactness of the imitation site soil, thus improving the capillary action and disintegration resistance of the imitation site soil.

**Figure 10:**
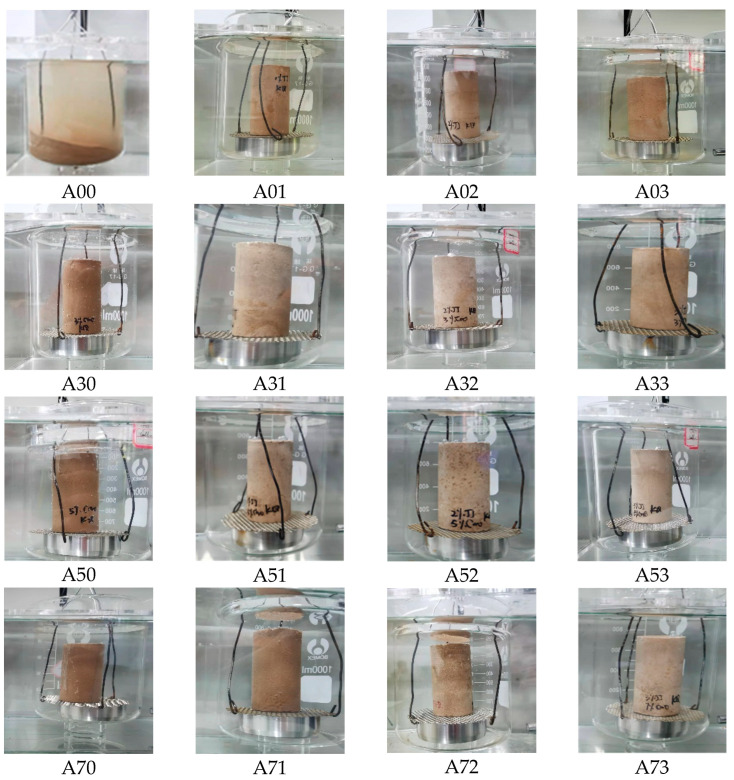
Appearance change of each ratio specimen after 24 h of disintegration.

During the disintegration test, only specimen A00 disintegrated and became a pile of loose soil, so it was impossible to weigh its mass to calculate its disintegration rate. None of the other compound specimens disintegrated and they only slightly absorbed water. The two materials had obvious effects on improving the disintegration resistance of the imitation site soil. In order to express the disintegration resistance and water stability of different ratio combinations more accurately, the recorded data were plotted against the disintegration test time as the horizontal coordinate, and the mass water absorption rate as the vertical coordinate, as shown in Figure 10.

**Figure 11:**
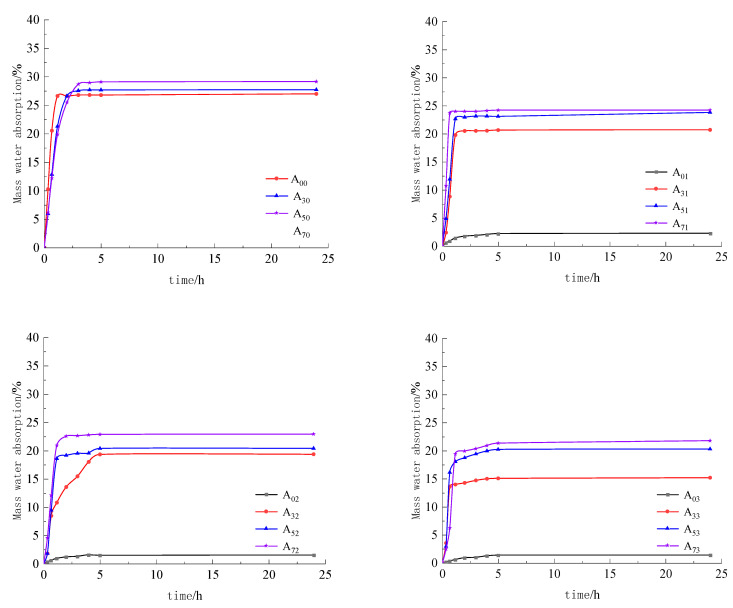
Relationship between disintegration time and mass water absorption curve.

From Figure 11, it can be seen that, with the increase in lime admixture, the mass water absorption of the specimen increases slowly, which is because it takes a longer time for the lime to be completely carbonized into CaCO_3_ [25], and at this time, part of the lime inside the specimen is not carbonized into CaCO_3_ precipitation, and the residual Ca(OH)_2_ will increase the capillary effect of the imitation site soil. With the increase in waterproofing material, the water absorption of the specimen’s mass gradually decreased; it can be seen that waterproofing materials are more effective in improving the water resistance of the imitation site soil. The overall water absorption rate of the specimen’s mass mixed with only water repellent material is lower, compared to the specimen’s mass absorption rate without water repellent material, which is reduced in the range of 2.76–27.77%. When lime and water repellent materials were incorporated at the same time, the water resistance of the soil was slightly affected because the CO_2_ concentration in the maintenance environment was insufficient, which affected the chemical reaction between the water repellent materials and the lime in the soil; the effect of the maintenance environment on the water resistance of the soil could be investigated in the next stage of the experiment. 

### 3.3. Mechanism of the Water Resistant Action of Reinforcement Material Modified Soil Sites

The properties of soil sites in the Henan section of the Yellow River Basin are mostly powder clay, which has the characteristics of easy water absorption and weathering, vigorous capillary action, and fast salt transport. Capillary water can have a large deterioration effect on the soil site, and the physical–chemical interaction between the water molecules and soil particles leads to changes in the physical properties of the soil’s skeleton and the pore water, resulting in dry shrinkage and cracking of the soil site, a reduction of free energy, and the destruction of the internal structure of the soil site, which leads to the reduction in the strength of the soil site.

**Figure 12:**
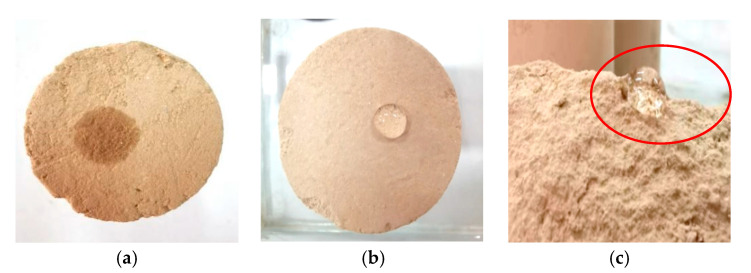
Water droplet pattern on the soil surface of the site before and after consolidation. (**a**) A00Surface. (**b**) A73Surface. (**c**) A73Internal.

The morphology of water droplets on the surface of the site soil is shown in Figure 12. Figure 12a A00 surface water droplets quickly penetrated into the soil sample, the contact angle was less than 90°, and showed good hydrophilicity; Figure 12b A73 surface water droplets did not experience infiltration phenomenon, the contact angle was greater than 90°, the water molecules did not easily enter the capillary inside the specimen, there was better water and moisture resistance, etc.; Figure 12c A73 inside the specimen had the same water resistance as the specimen’s surface, water droplets self-agglomerated into clusters, there was a large reduction in surface tension, the water contact area with the specimen was smaller, the surface free energy of the reinforced soil site was greatly reduced, and the water resistance was significantly improved. There was a large reduction in surface tension, the water and specimen contact area was small, the water molecules did not penetrate, the surface free energy of the reinforced soil site was greatly reduced, and the water resistance had been significantly improved. Waterproofing materials are dewatered and condensed inside the soil site to form a water-repellent grid structure film, and the surface of this methylsilicate film is rough and uneven [26], which can effectively reduce the capillary action of the soil site, enhance the interparticle adhesion of the soil site, and to a certain extent, form an active water barrier to prevent the diffusion of water molecules into the interior of the specimen, which is conducive to improving the mechanical properties and water resistance of the soil site. This is because the polysiloxane film can enhance the water resistance and disintegration resistance of the sample.

### 3.4. Micro-Analysis

In order to observe the changes in particle morphology and pore characteristics inside the modified specimens, SEM was used to analyze the specimens A00, A03, A70 and A73 after 1000 times magnification to further investigate the mechanism of the improved shear strength, shear strength index and disintegration resistance of the modified materials used to improve the imitation site soil.

**Figure 13:**
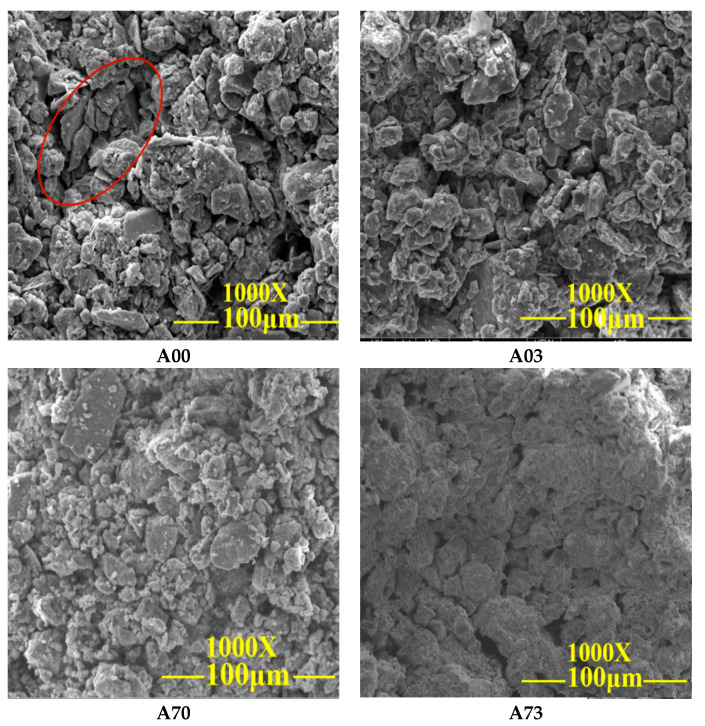
SEM image before and after specimen modification.

Figure 13 shows the specimen before and after the improvement SEM diagram; as seen in the figure: the A00 specimen’s internal soil particle porosity is larger, the soil particles are not uniformly arranged, and the particle gradation is not good. There is less adhesive on the surface of soil particles, resulting in weak adhesion between particles. The A03 specimen’s internal structure porosity has slightly improved, and the waterproof material generated a small amount of silicate gel particles, which can play a filling role in the powder clay; however, the overall particle arrangement and particle gradation improvement is not obvious. The A70 specimen was more improved than the A00 and A03 specimens and the pore characteristics are more closely connected; the particle gradation improvement is also better. The chemical reaction between the lime and the Ca(OH)_2_ crystals and CaCO_3_ precipitates inside the imitation site soil filled the micro-pores and micro-cracks between the soil particles. In addition, the pore structure of the soil particles in specimen A73 was most obviously improved, and the soil particles were more closely connected to each other, the Ca(OH)_2_ crystals, and the CaCO_3_ precipitation, which can improve the shear strength, cohesion and internal friction angle of the specimens.

## 4. Conclusions

Through the study of this paper, the following conclusions can be obtained:

(1) The shear strengths of the specimens with different ratio combinations under normal stresses of 50 kPa, 100 kPa, 150 kPa and 200 kPa were improved to different degrees compared with A00, ranging from 10.53–75% under 50 kPa, 4.17–46.53% under 100 kPa, 6.71–39.02% under 150 kPa and 10.29–56% under 200 kPa. Both lime and waterproofing materials are beneficial to the improvement of the shear strength of soil sites, and the effect of lime is greater than that of waterproofing materials.

(2) With the increase in lime and waterproofing materials, the overall trend of the cohesion and internal friction angle of the imitation site soil increased. The cohesive force and internal friction angle of the imitation site soil increased when the lime mixture was 3%, 5% and 7%, and the cohesive force of the imitation site soil increased at the maximum rate of 59.23% when the water repellent material was 2% and the lime mixture was 7%; the internal friction angle of the imitation site soil varied in the range of 36.72°–41.61° when the lime and water repellent materials were used to improve the imitation site soil at the same time. The variation is small.

(3) The soil group placed in static water disintegrated within a short period of time; the water stability and disintegration resistance was poor. In the other ratio specimens, no disintegration phenomenon occurred, and the water stability and disintegration resistance performance was good. During the disintegration test process, there was slight water absorption, which led to the quality of the specimen being increased, not reduced. Only when the specimens were mixed with waterproof material was the surface smooth and complete, with no disintegration phenomena, and the water absorption phenomena was not obvious, indicating that the waterproof material improves the crumbling resistance of imitation site soil performance and has more effective water stability. When the specimens were only mixed with lime, during the disintegration test process there were tiny bubbles, indicating that the sample had a small amount of water absorption phenomena; however, an overall collapse did not occur, and the disintegration resistance was good.

(4) The most obvious improvement in the internal pore structure of the modified imitation site soil is the tighter connection between the soil particles and the surface of the specimen, which had more obvious flocculation. This is due to the chemical reaction of lime inside the specimen generating Ca(OH)_2_ crystals and CaCO_3_ precipitation, which can improve the shear strength, cohesion and internal friction angle of the specimen.

## Figures and Tables

**Table 1:** Material and element composition of silty clay.

Chemical Compound	Content (%)	Element	Content (%)	Chemical Compound	Content (%)	Element	Content (%)
SiO_2_	57.79	Si	27.02	ZrO_2_	0.019	Zr	0.0141
Al_2_O_3_	16.05	Al	8.49	Rb_2_O	0.0132	Rb	0.011
CaO	10.52	Ca	7.52	Cr_2_O_3_	0.0161	Cr	0.0121
Fe_2_O_3_	6.16	Fe	4.31	NiO	0.0063	Ni	0.0072
MgO	3.11	Mg	1.89	CuO	0.0058	Cu	0.005
K_2_O	1.5	K	1.13	As_2_O_3_	0.003	As	0.0046
Na_2_O	1.49	Na	1.11	I	0.0046	I	0.0049
TiO_2_	0.815	Ti	0.488	Co_3_O_4_	0.0035	Co	0.0041
P_2_O_5_	0.214	Px	0.32	PdO	0.0056	Pd	0.0046
MnO	0.117	Mn	0.0933	TeO_2_	0.0051	Te	0.0033
SO_3_	0.0544	Sx	0.0907	Ga_2_O_3_	0.0032	Ga	0.0026
SrO	0.0304	Sr	0.0218	WO_3_	0.0034	W	0.0027
ZnO	0.0228	Zn	0.0257	PbO	0.0031	Pb	0.0024
V_2_O_5_	0.0207	V	0.0183	Sc_2_O_3_	0.0017	Sc	0.0029
Cl	0.02	Cl	0.0116	RuO_4_	0.0014	Ru	0.0023

**Table 2:** Specimen mix ratio design.

Mixing Ratio	Lime/%	Waterproof Material/%	Mixing Ratio	Lime/%	Waterproof Material/%
A00	0	0	A50	5	0
A01		1	A51		1
A02		2	A52		2
A03		3	A53		3
A30	3	0	A70	7	0
A31		1	A71		1
A32		2	A72		2
A33		3	A73		3

## Data Availability

The data used to support the findings of this study are included within the article.
